# Whole Genome Sequencing Identifies a 78 kb Insertion from Chromosome 8 as the Cause of Charcot-Marie-Tooth Neuropathy CMTX3

**DOI:** 10.1371/journal.pgen.1006177

**Published:** 2016-07-20

**Authors:** Megan H. Brewer, Rabia Chaudhry, Jessica Qi, Aditi Kidambi, Alexander P. Drew, Manoj P. Menezes, Monique M. Ryan, Michelle A. Farrar, David Mowat, Gopinath M. Subramanian, Helen K. Young, Stephan Zuchner, Stephen W. Reddel, Garth A. Nicholson, Marina L. Kennerson

**Affiliations:** 1 Northcott Neuroscience Laboratory, ANZAC Research Institute, Concord, New South Wales, Australia; 2 Sydney Medical School, University of Sydney, Camperdown, New South Wales, Australia; 3 Discipline of Pathology, University of Sydney, Camperdown, New South Wales, Australia; 4 The Institute for Neuroscience and Muscle Research, The Children’s Hospital at Westmead, Westmead, New South Wales, Australia; 5 T.Y. Nelson Department of Neurology and Neurosurgery, The Children’s Hospital at Westmead, Westmead, New South Wales, Australia; 6 Paediatrics and Child Health, University of Sydney, Camperdown, New South Wales, Australia; 7 Department of Neurology, Royal Children’s Hospital, Parkville, Victoria, Australia; 8 Murdoch Childrens Research Institute, Parkville, Victoria, Australia; 9 Department of Paediatrics, University of Melbourne, Parkville, Victoria, Australia; 10 Department of Neurology, Sydney Children’s Hospital, Randwick, New South Wales, Australia; 11 School of Women’s and Children’s Health, UNSW Medicine, University of New South Wales, Kensington, New South Wales, Australia; 12 Department of Medical Genetics, Sydney Children’s Hospital, Randwick, New South Wales, Australia; 13 Department of Paediatrics, John Hunter Children’s Hospital, Newcastle, New South Wales, Australia; 14 Department of Paediatrics, Royal North Shore Hospital, St Leonards, New South Wales, Australia; 15 Northern Clinical School, Sydney Medical School, University of Sydney, St Leonards, New South Wales, Australia; 16 Department of Neurogenetics, The Children’s Hospital at Westmead, Westmead, New South Wales, Australia; 17 John P. Hussman Institute for Human Genomics, University of Miami Miller School of Medicine, Miami, Florida, United States of America; 18 Dr. John T. Macdonald Department of Human Genetics, University of Miami Miller School of Medicine, Miami, Florida, United States of America; 19 Department of Neurology, Concord Repatriation General Hospital, Concord, New South Wales, Australia; 20 Molecular Medicine, Concord Repatriation General Hospital, Concord, New South Wales, Australia; Pennsylvania State University, UNITED STATES

## Abstract

With the advent of whole exome sequencing, cases where no pathogenic coding mutations can be found are increasingly being observed in many diseases. In two large, distantly-related families that mapped to the Charcot-Marie-Tooth neuropathy CMTX3 locus at chromosome Xq26.3-q27.3, all coding mutations were excluded. Using whole genome sequencing we found a large DNA interchromosomal insertion within the CMTX3 locus. The 78 kb insertion originates from chromosome 8q24.3, segregates fully with the disease in the two families, and is absent from the general population as well as 627 neurologically normal chromosomes from in-house controls. Large insertions into chromosome Xq27.1 are known to cause a range of diseases and this is the first neuropathy phenotype caused by an interchromosomal insertion at this locus. The CMTX3 insertion represents an understudied pathogenic structural variation mechanism for inherited peripheral neuropathies. Our finding highlights the importance of considering all structural variation types when studying unsolved inherited peripheral neuropathy cases with no pathogenic coding mutations.

## Author Summary

Next generation sequencing technologies have greatly advanced disease gene discovery for Charcot-Marie-Tooth (CMT) disease and related inherited peripheral neuropathies. However, many families with CMT remain unsolved after all protein-coding sequences have been interrogated through whole exome sequencing. The pathogenic mutations in these unsolved families may be non-coding point mutations, small indels or large structural variations involving thousands to millions of base pairs. In two large, distantly related families with X-linked CMT, all known protein-coding sequence variants were tested and no causal variant was found. Using whole genome sequencing we identified a 78 kb 8q24.3 insertion at chromosome Xq27.1 as the likely underlying cause of neuropathy in these two families. This is the first report of a large insertion causing CMT and highlights an understudied disease mechanism for inherited peripheral neuropathy.

## Introduction

Charcot-Marie-Tooth (CMT) disease is the collective name given to a group of clinically and genetically heterogeneous inherited peripheral neuropathies that affect both motor and sensory neurons. Over 80 genes have been associated with CMT and other related inherited peripheral neuropathies, which account for up to 80% of CMT cases [1–4]. In our Australian cohort, after extensive whole exome sequencing (WES) analysis of multiple family members, a proportion of these unsolved families also have no detectable protein-coding mutation in the exome. This suggests that point mutations and small insertions/deletions of non-coding DNA and DNA structural variations may account for some of the unsolved cases.

CMTX3, a subtype of X-linked CMT, is one such locus which has remained unsolved after extensive molecular analyses. The CMTX3 locus was initially mapped to the long arm of chromosome X in two American families [5]. The locus was confirmed and refined to a 5.7 Mb region on chromosome Xq26.3-q27.3 in a large United Kingdom/New Zealand family (CMT623) [6] and an Australian family (CMT193-ext) [7]. Affected males from these two families generally presented a slightly milder phenotype than the more common X-linked CMT subtype, CMTX1. However the degree of severity varied. Onset of disease generally started in the first decade, initially presenting in the lower limbs. Sensory symptoms included marked pain and paraethesia in hands and feet as well as sensory loss. Tremor in hands and spastic paraparesis was not observed. Nerve conduction velocities data suggested these patients have an intermediate CMT. Female carriers were considered asymptomatic with normal nerve conduction velocities, however the observation of subtle clinical signs including high-arched feet, weakness in foot dorsiflexion and loss of ankle reflexes suggested female carriers may present very mild symptoms [6].

The two families carry the same CMTX3 haplotype, suggesting they share an identical genetic mutation inherited from a common ancestor. Genotype analysis of one of the original American families (US-PED2) initially suggested this family also carried the distal portion of the CMTX3 haplotype [7]. However, re-examination of family US-PED2 by whole exome sequencing (WES) identified a known *BSCL2* mutation (c.263A>G, p.Asn88Ser) as the genetic cause of disease in the family [8]. Mutation screening families CMT623 and CMT193-ext excluded all coding sequences mapping within the 5.7 Mb locus for pathogenic mutations [6, 9]. Therefore, we employed whole genome sequencing (WGS) to interrogate the disease locus for pathogenic non-coding single nucleotide variants and structural variations in these families.

## Results/Discussion

Two affected males and an unaffected male control from each of the families CMT623 and CMT193-ext (i.e. four patients and two controls) underwent WGS. An average of 134 Gb of sequence was generated for each individual. On average, 96% of total reads mapped to the reference genome and all samples had a minimum depth of coverage (DOC) of 44X across the whole genome (Table 1). The CMTX3 locus had an average DOC of 24X, which reflected the males being hemizygous for chromosome X.

**Table 1:** Whole genome sequence quality data analysis.

	Individual	Total Reads	Mapped Reads	% Mapped Reads	Average Coverage Genome	Average Coverage ChrX	MapQ Score
**CMT193**	Affected 1	1,767,871,080	1,745,034,895	99	56.3	29.4	52.3
	Affected 2	1,398,881,966	1,377,234,225	98	44.4	22.8	51.6
	Control 1	1,415,254,852	1,389,518,446	98	44.8	23.4	51.5
**CMT623**	Affected 3	1,404,391,674	1,375,198,612	98	44.5	23.2	51.5
	Affected 4	1,423,140,198	1,372,077,383	96	44.3	23.1	49.4
	Control 2	1,419,371,166	1,392,511,028	98	45.1	23.3	51.7

Patient and control sequence alignments revealed the presence of split-reads at Xq27.1 (Table 2). The four affected males consistently showed split reads at the genomic location chrX:139,502,948. The corresponding paired ends for the split reads mapped both upstream and downstream of the suggestive breakpoint at chromosome Xq27.1. Split-reads at this location were not identified in the two unaffected males. The unaligned sequences of these split-reads mapped to two genomic regions (chr8:145,768,312 and chr8:145,848,158). These genomic positions are located 78 kb apart on chromosome 8q24.3 and represent the boundaries of the DNA region that has been inserted into chromosome Xq27.1 in the CMTX3 patients. Patient WGS data also showed split-reads on chromosome 8 that contained Xq27.1 sequence and paired with reads anchoring to these two locations on chromosome 8q24.3. Further analysis also identified discordant paired ends in which one read pair mapped to Xq27.1 and the other read pair mapped to 8q24.3. This was observed in all four patients and absent from the two control samples. Table 2 summarizes the number of split-reads and discordant paired ends identified for each patient. Based on these data we predicted that a 78 kb sequence from 8q24.3 had been inserted into chromosome Xq27.1 in CMTX3 patient DNA.

**Table 2:** Split-reads and discordant paired ends mapping to Xq27.1 and 8q24.3 in whole genome sequencing data from affected males.

	Individual	Read Type	Proximal Breakpoint	Distal Breakpoint
**CMT193**	Affected 1	Split-reads Paired end maps to chrX	7	9
		Split-reads Paired end maps to chr8	7	6
		Discordant paired ends	14	15
	Affected 2	Split-reads Paired end maps to chrX	8	7
		Split-reads Paired end maps to chr8	3	5
		Discordant paired ends	10	11
**CMT623**	Affected 3	Split-reads Paired end maps to chrX	7	13
		Split-reads Paired end maps to chr8	4	6
		Discordant paired ends	15	16
	Affected 4	Split-reads Paired end maps to chrX	6	6
		Split-reads Paired end maps to chr8	3	6
		Discordant paired ends	8	12

**Fig 1:**
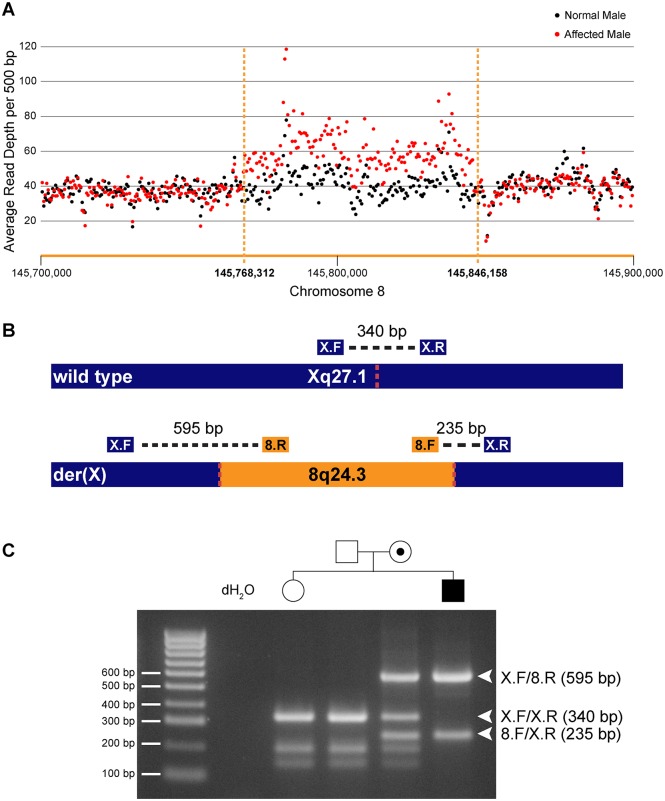
Identification and confirmation of a 78 kb chromosome 8q24.3 insertion in patients with CMTX3. (A) Whole genome sequencing depth of coverage for affected (red) and normal (black) males across the 8q24.3 insertion and flanking sequence. (B) Depiction of wild type chromosome X (top) and mutant chromosome X (bottom). The location of primers and amplicon sizes for the multiplex PCR genotyping assay are shown. Dotted red lines represent insertion breakpoints. (C) Size fractionation of multiplex PCR genotyping assay for a subset of family members from CMT623. Individual genotypes are depicted above the gel lane. Expected band sizes for the various primer combinations are listed to the right. Unaffected hemizygous males and homozygous females generate a single 340 bp amplicon; affected hemizygous males generate 595 bp and 235 bp amplicons crossing the proximal and distal breakpoints, respectively; carrier females amplify all three amplicons.

To determine whether the entire 78 kb region from chromosome 8q24.3 had been duplicated and inserted into Xq27.1 we assessed the DOC across the genomic interval chr8:145,700,000–145,900,000 (Table 3). Control males showed a uniform DOC across the entire 200 kb region with a mean DOC of 40X. The affected males, however, showed a 1.6-fold increase in DOC (mean DOC of 64X) within the boundaries of the insertion breakpoints (chr8:145,768,312–145,846,158). The DOC for the genomic regions immediately flanking the 8q24.3 insert sequence were similar to the controls (Fig 1A). These data suggested that patients with CMTX3 carry an extra copy of the 78 kb region from chromosome 8q24.3 through the interchromosomal insertion event at the CMTX3 locus.

**Table 3:** Average depth ± SD of sequence coverage across 8q24.3.

	CMT193-ext			CMT623		
Genomic Region	Affected 1	Affected 2	Control 1	Affected 3	Affected 4	Control 2
Upstream^a^	45 (±7)	36 (±6)	34 (±6)	36 (±6)	36 (±6)	37 (±5)
Insert^b^	76 (±14)	61 (±11)	39 (±8)	60 (±12)	60 (±11)	41 (±8)
Downstream^c^	50 (±8)	41 (±7)	39 (±7)	39 (±7)	40 (±7)	40 (±7)

^a^—chr8:145,700,000–145,768,311

^b^—chr8:145,768,312–145,846,158

^c^—chr8145,846,159–145,900,000

We next assessed whether the interchromosomal insertion segregated with the disease in our two distantly related families using a multiplex PCR genotyping assay (Fig 1B). Genotyping results for a subset of family members from CMT623 are shown (Fig 1C). The different sized amplicons were confirmed via Sanger sequencing (S1 Fig). The 78 kb insertion segregated in 55 individuals (25 affected males and 30 carrier females) from families CMT623 and CMT193-ext. The 78 kb insertion was not seen in the 50 unaffected members (30 males, 20 females) from families CMT623 and CMT193-ext that were available for testing. All individuals were clinically diagnosed and genotyped for the CMTX3 haplotype prior to this study. The 8q24.3 interchromosomal insertion was absent in 627 control X chromosomes from neurologically normal females (n = 252) and males (n = 123).

**Fig 2:**
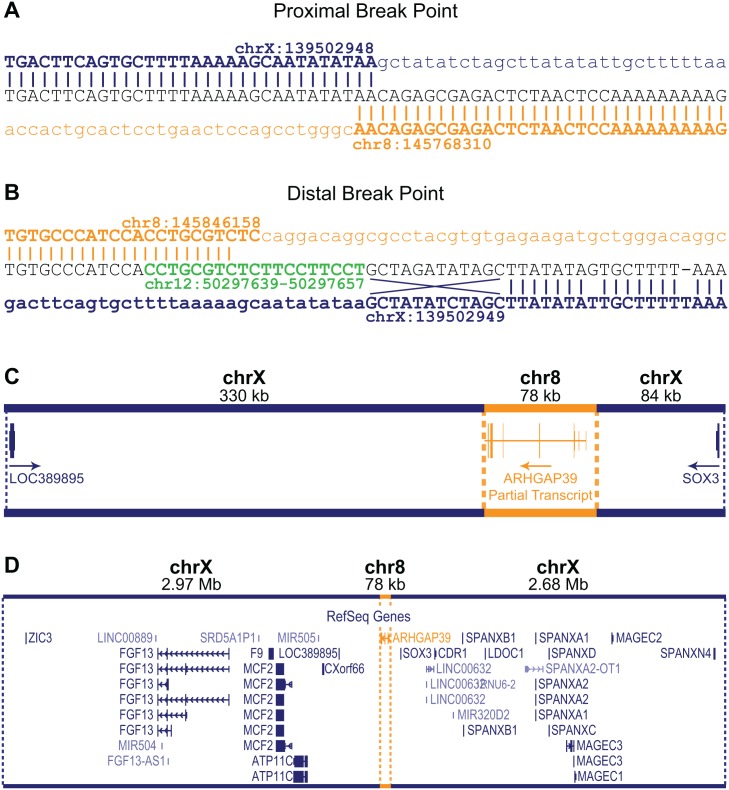
Characterization of the CMTX3 insertion. Sequence analysis of the proximal (A) and distal (B) breakpoints. Reference sequence for chromosome X and chromosome 8 are indicated in blue and orange, respectively. The distal breakpoint includes additional sequence from chromosome 12 (in green) and small rearrangements of the chromosome X sequence including an inversion of 12 bp, and a base pair substitution and a base pair deletion. (C) The 78 kb 8q24.3 sequence (in orange) contains the partial 5’*ARHGAP39* transcript which has been inserted 330 kb downstream and 84 kb upstream of the genes *LOC389895* and *SOX3*, respectively (in blue). The direction of transcripts are indicated by the arrow. (D) Location of the 78kb 8q24.3 insertion sequence (in orange) relative to the whole of the 5.7 Mb CMTX3 locus (in blue).

Sanger sequencing the amplicons spanning the insertion breakpoints confirmed the WGS predictions (Fig 2A and 2B). The 8q24.3 sequence inserted directly between the genomic locations chrX:139,502,948–139,502,949. For the proximal breakpoint, the exact location of the end sequence from chromosome X and start position of the 8q24.3 insertion sequence could not be unambiguously defined due to a 2 bp overlap (AA) in the sequence (Fig 2A). For the purposes of defining breakpoints, we have designated the chromosome 8 insertion start position as chr8:145,768,312. The distal breakpoint is more complex (Fig 2B). The 8q24.3 insertion sequence ends at position chr8:145,848,158 followed by a small insertion from chromosome 12q13.12, which maps within an intron of the *FAIM2* gene. A total of 19 bp from the small insertion sequence maps to 12q13.12 however the first 10 bp also overlap with chromosome 8 (green sequence, Fig 2B). Adjacent to the 12q13.12 insertion, the first 12 bps of chromosome X at the distal breakpoint are inverted. There is also a single nucleotide variant (T>G) at chrX:139,502,968 and a single nucleotide deletion at chrX:139,502,976 (Fig 2B). These variants appear to be unique to the two CMTX3 families and have not been reported in variant databases including the 1000 Genomes Project [10] or dbSNP [11].

The 8q24.3 insertion region is 77,856 bp and contains a partial transcript of the *ARHGAP39* gene (exons 1–7) encoded on the negative strand (Fig 2C). The duplicated 8q24.3 sequence has inserted into an intergenic region of Xq27.1 with the nearest flanking genes being *LOC389895* (located 329 kb downstream proximal to the 78 kb insertion) and *SOX3* (located 84 kb distal to of the insertion) (Fig 2C and 2D).

Based on the genomic architecture of the CMTX3 interchromosomal insertion, we hypothesized two possible mechanisms that could lead to peripheral neuropathy: 1) overexpression of the partial *ARHGAP39* transcript due to 8q24.3 trisomy; or 2) transcriptional dysregulation of one or more genes mapping within the CMTX3 locus.

Aberrant splicing with the *ARHGAP39* partial transcript may also be a possible mechanism. However this is unlikely, as the inserted *ARHGAP39* partial transcript is predicted to be transcribed on the negative strand and the nearest downstream gene, *LOC389895*, is a single exon gene transcribed from the positive strand (Fig 2C).

Copy number variations (CNVs) that result in the duplication or deletion of a gene is a well-known cause of CMT neuropathy, indicating that peripheral nerves are sensitive to gene dosage. A 1.5 Mb duplication on chromosome 17p12 [12, 13], resulting in trisomy of the *PMP22* gene [14–17], causes the most common form of CMT (CMT1A). This was the seminal example of a CNV causing disease. The reciprocal 1.5 Mb 17p12 deletion causes hereditary neuropathy with liability to pressure palsies (HNPP) [18]. Although relatively rare [19–21], a small number of individual cases describing whole and partial gene duplications or deletions for other CMT loci including *MPZ* [21–23], *GJB1* [24–26], *MFN2* [27], and *NDRG1* [28] have also been reported. Currently there are no interchromosomal insertions reported as a cause of CMT.

**Fig 3:**
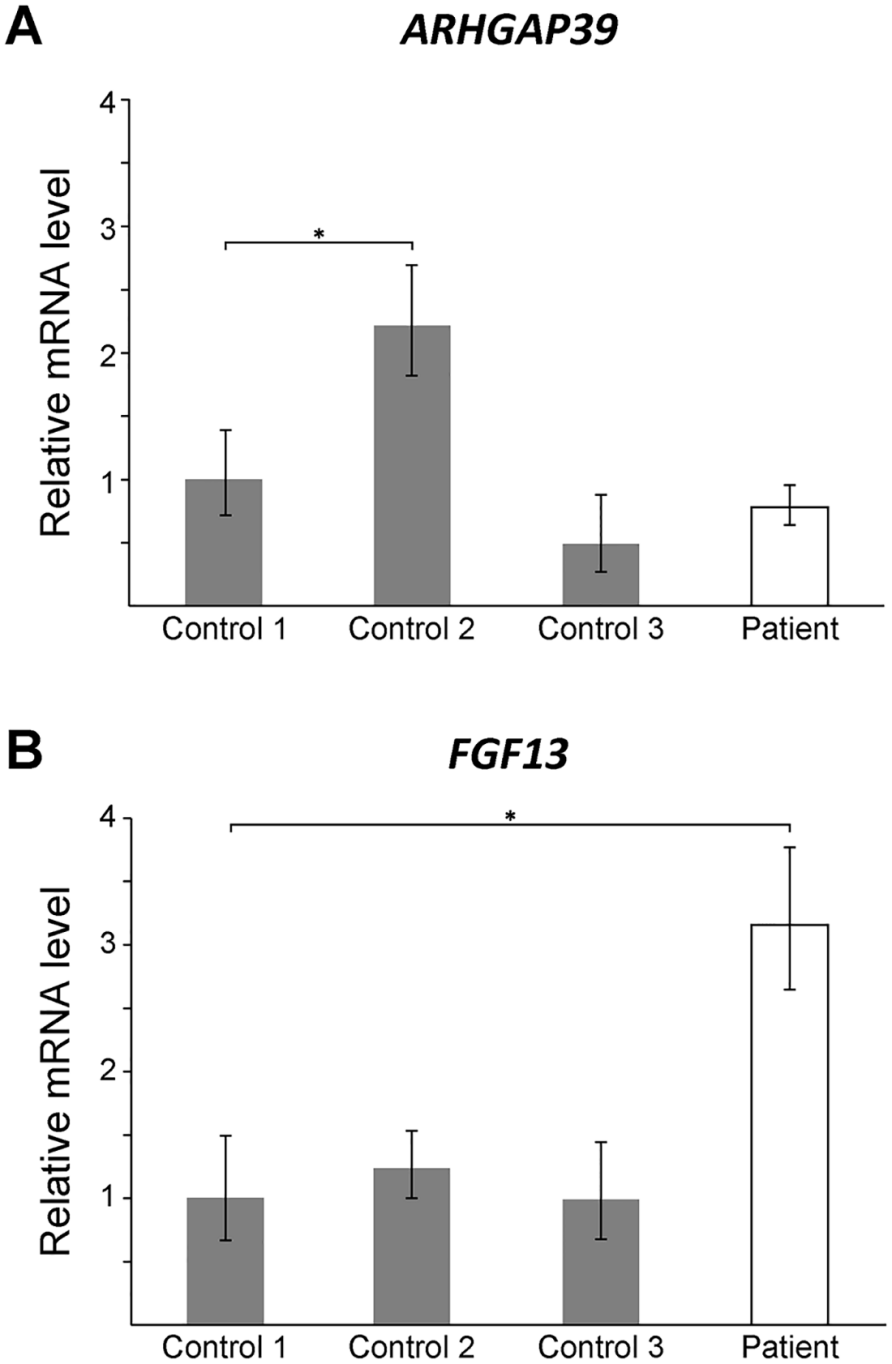
*FGF13* mRNA expression is elevated in patient lymphoblasts. Quantitative RT-PCR showing mRNA levels for *ARHGAP39* (A) and *FGF13* (B) from patient lymphoblasts relative to three normal controls. Bars show the mean mRNA levels (± SD; error bars) relative to Control 1, which has been set to +1. A student t-test was performed comparing each value to Control 1 (*, p < 0.05).

To assess whether the CMTX3 insertion affects gene expression, quantitative RT-PCR analysis was used to assess the mRNA expression levels of candidate genes in patient and control lymphoblasts. No difference in *ARHGAP39* expression was observed between the patient and controls (Fig 3A). This suggested that trisomy of the *ARHGAP3* partial transcript is unlikely the underlying cause of neuropathy.

Large rearrangements disrupting non-coding DNA sequences are likely to cause disease by dysregulating the transcriptional expression of one or more nearby genes [29]. Duplication of a 186 kb sequence located 3 kb distal to the *PMP22* gene [30, 31], harboring Schwann cell-specific transcription factor binding sites [32], was found to cause CMT1A by dysregulating *PMP22* expression [30, 31]. Non-coding DNA structural variations can disrupt the interaction between a gene and its functional non-coding DNA sequences (such as promoters, enhancers and silencers) or introduce new interactions, resulting in dysregulated temporal and spatial gene expression [29, 33, 34]. Recent studies have shown that regulatory elements and their target genes cluster within local chromatin interaction domains or “topologically associated domains” [35]. Genomic rearrangements that physically disrupt the boundaries of these domains introduce ectopic interactions between regulatory elements and genes that can cause disease [29]. However, based on Hi-C profile data from human embryonic stem cells [35] the 78 kb sequence from 8q24.3 appears to have inserted into a topologically associated domain without disrupting the boundaries (S2 Fig) suggesting that if the CMTX3 mutation dysregulates a nearby gene it is likely through some other mechanism.

To explore the possible mechanism of transcriptional dysregulation of one or more genes mapping within the CMTX3 locus, we assessed the expression of *SOX3* and *FGF13*. Large DNA interchromosomal insertions at the Xq27.1 locus have been previously reported to cause a range of phenotypes [36–40] and these two genes are known to be dysregulated in patients with other Xq27.1 interchromosomal insertions [38, 40].

*SOX3* encodes the sex determining region Y-box 3 transcription factor. In an XX sex reversal patient carrying a 774 kb interchromosomal insertion from chromosome 1q25.3, an increase in *SOX3* expression was observed in the patient lymphoblasts [40]. *SOX3* expression however was not detected in the control lymphoblasts. In both our patient and control lymphoblast cell lines, *SOX3* mRNA expression could not be detected (S3 Fig). These results reflect previous reports of *SOX3* expression in control lymphoblasts [40] and it is likely that *SOX3* is silenced by methylation in lymphoblasts [41]. Unlike the 1q25.3 interchromosomal insertion, the presence of the 8q24.3 interchromosomal insertion does not appear to affect *SOX3* expression in lymphoblasts.

*FGF13* encodes the fibroblast growth factor 13 protein that is part of the fibroblast growth factor homologous family [42]. Hypertrichosis patients carrying a 389 kb interchromosomal insertion from chromosome 6p21.1 showed reduced *FGF13* expression in patient hair follicles [38]. We observed a 3-fold increase in expression in lymphoblast cells from the CMTX3 patient (Fig 3B). Although the assay could not distinguish between the different *FGF13* isoforms, our preliminary finding demonstrates that the 8q24.3 interchromosomal insertion dysregulates *FGF13* expression in CMTX3 patient lymphoblasts. We hypothesize that if similar dysregulation of *FGF13* gene expression were to be observed in patient neurons this could be the underlying cause of disease in CMTX3 patients. It is also possible that the observed dysregulation of *FGF13* is a benign, bystander effect of the 78 kb interchromosomal insertion. Further gene expression studies on *FGF13* and the remaining genes mapping to the CMTX3 locus, will be required to fully determine the pathogenic consequence of the CMTX3 8q24.3 insertion.

**Fig 4:**
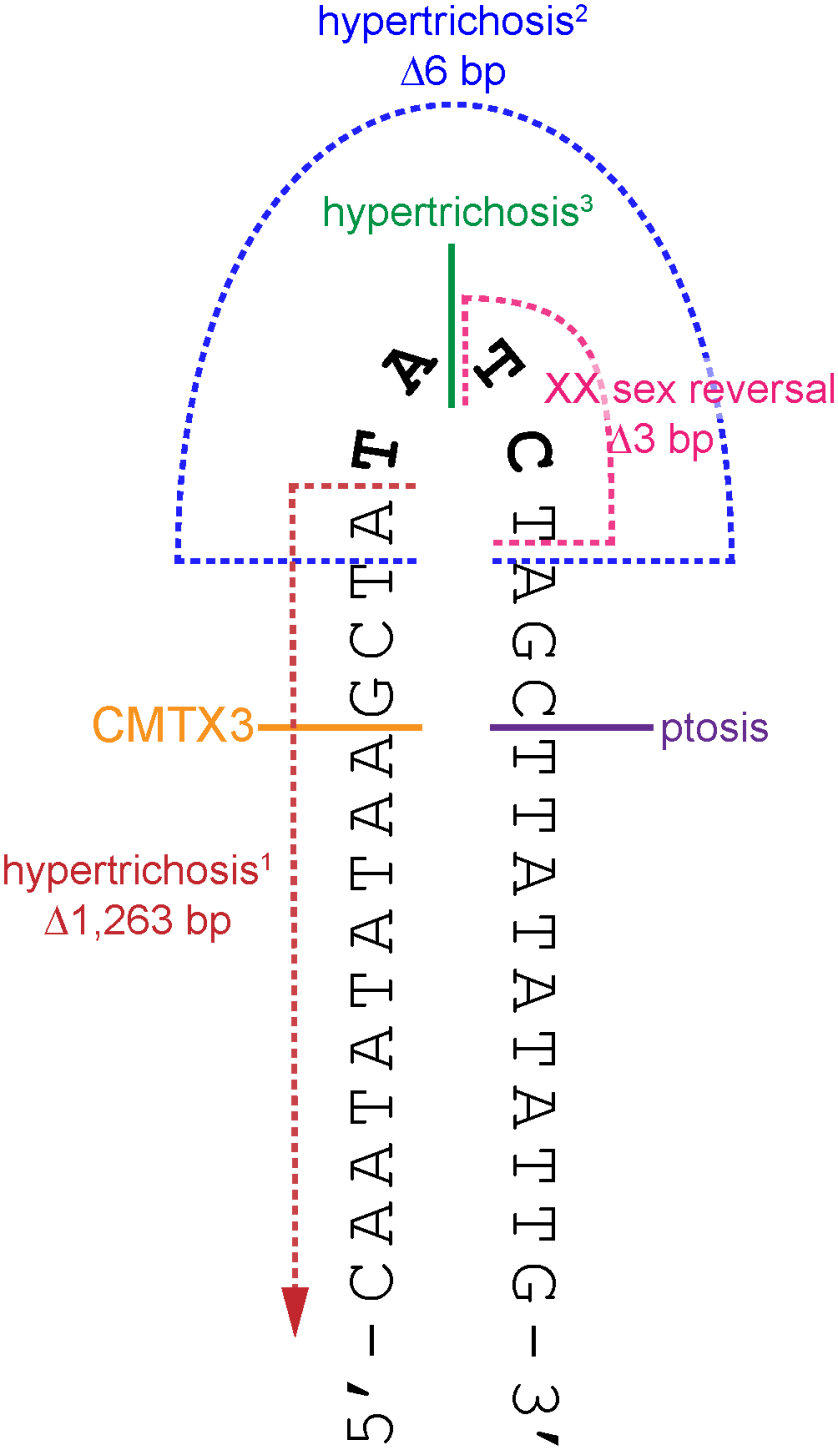
The breakpoints of disease-associated interchromosomal insertions at Xq27.1 localize near the center of 180 bp palindrome sequence. Cartoon depicts a portion of the palindrome sequence (chrX:139,502,939–139,502,970) with the positive strand folded upon itself in a hairpin loop (black). The four non-palindromic bases in the middle of the 180 bp sequence (TATC, bolded black) are predicted to form the head of the hairpin loop. The locations of the breakpoints on chromosome Xq27.1 for CMTX3 (orange); hypertrichosis^1^ (red, [37]); hypertrichosis^2^ (blue, [37]); hypertrichosis^3^ (green,[38]); ptosis (pink; Bunyan [39]); and XX sex reversal (purple, Haines [40]) are marked out on the hairpin structure. Single breakpoints are depicted by a solid line. Multiple breakpoints are indicated by broken lines.

There have been six large interchromosomal insertions previously reported; each originating from unique genomic regions and ranging from 124–774 kb [36–40]. These interchromosomal insertions have been shown to cause hypoparathyroidism [36], hypertrichosis [37, 38], ptosis [39], and XX male sex reversal [40]. CMTX3 is the fifth disease phenotype to be associated with an Xq27.1 interchromosomal insertion, clearly suggesting there is a recurrent mutation mechanism at the Xq27.1 locus. There are several mutation mechanisms that give rise to structural variations (recently reviewed in [43, 44]). We propose that this recurring mutation mechanism is possibly due to double stranded DNA breaks occurring in the 180 bp palindrome sequence at Xq27.1 [37] followed by incorrect repair of the DNA break through microhomology-mediated break-induced replication [45, 46]. For most of the interchromosomal insertions, including the CMTX3 insertion, at least one breakpoint is located near the center of the 180 bp palindrome sequence, close to where the hairpin loop is predicted to form (Fig 4) [37–40]. Hairpin loops are susceptible to double stranded DNA breaks due to endonuclease activity and are common hotspots for translocations [47]. Since the chromosome X breakpoints of these interchromosomal insertions localize within this hairpin structure, this suggests that hairpin formation of the palindrome sequence and endonuclease activity may be the initial process of the recurrent mutation mechanism.

Microhomology-mediated break-induced replication (MMBIR) coupled with fork stalling and template switching (FoSTeS) has been proposed as an alternative model for the formation of genomic rearrangements that cannot be explained by non-allelic homologous recombination [45, 48, 49]. In this model, microhomology-induced template switching occurs where nearby single-stranded DNA is used as template to repair DNA breaks. Depending on the template, this results in the formation of deletions, duplications, triplications inversions or translocations that are flanked by minimal sequence homology of 2–6 bp at the breakpoints [45]. Further complexity at the genomic rearrangement breakpoints, involving small deletions and/or small insertions of unlinked or unknown sequences, are also commonly observed and is likely due to multiple template-switching events occurring during the repair process [49].

Sequencing the breakpoints of the CMTX3 rearrangement revealed an additional 19 bp from chromosome 12q13.12, an inversion of 12 bp from chromosome Xq27.1 and microhomology between chromosome X and chromosome 8 sequence as well as between the chromosome 8 and chromosome 12 sequence (Fig 2A and 2B). Microhomology, small deletions at the Xq27.1 sequence and additional small inserted sequences, from unlinked (i.e. from another chromosome) or unknown sources, also feature in the other disease-associated interchromosomal insertions at Xq27.1 [36–40] suggesting these insertions arose through MMBIR/FoSTeS.

Since each unique DNA insertion causes different disease phenotypes this suggests that the inserted genomic sequence is important. Based on the varying gene dysregulation observed for patients with hypertrichosis [38], XX sex reversal [40] and CMTX3, we predict the disease specificity from each interchromosomal insertion into Xq27.1 arises from the introduction of DNA regulatory elements that interact with the nearby genes in a tissue-specific manner. Unsolved Mendelian diseases mapping to the Xq27.1 region should therefore be assessed for large interchromosomal insertions using WGS analysis.

With 20% of our CMT families remaining genetically unsolved after WES [2], finding the causes of disease in these families is an important goal for inherited peripheral neuropathies. Our discovery suggests that structural variation involving non-coding DNA may explain a portion of the unsolved families. It also highlights the importance of looking beyond CNV when analyzing the genome for structural variation. Although the CMTX3 mutation represents trisomy of 8q24.3, given that this does not result in a dosage change for *ARHGAP39*, it is likely that the insertion itself underlies the peripheral neuropathy.

WGS provides a powerful tool to detect the full spectrum of DNA variation including all classes of structural variations [50, 51]. Given that structural variations are found throughout the general population [52, 53] distinguishing pathogenic and benign structural variations will be difficult without large families to confirm segregation. In time, improved annotation of benign genomic rearrangements in SV databases, that go beyond CNV and map the location and orientation of all SV subtypes, will assist in delineating pathogenic structural variations in patients. Pathogenic structural variations identified in families that are large enough for segregation analyses, as we have shown for the CMTX3 mutation, will provide genomic landmarks in which WGS data from smaller families can be mined for structural variation sequencing signatures (such as split reads and discordant paired ends). This strategy will, however, have limited use if structural variations causing inherited peripheral neuropathy prove to be rare private mutations. With decreasing WGS costs and improved sensitivity of WGS alignment algorithms, we predict that more structural variations are likely to be identified as the pathogenic cause of CMT. However, we acknowledge that the detection of these mutations in both the research and clinical diagnostic settings will be a challenge with no immediate solution.

In conclusion, we have provided compelling data supporting the likely genetic cause of CMTX3 neuropathy as a 78 kb interchromosomal insertion at Xq27.1 [der(X)dir ins(X;8)(q27.1;q24.3)]. Based on genealogy studies we believe this founder insertion originated prior to the early 1800s in a Scottish family. Our discovery is the first neuropathy caused by an Xq27.1 interchromosomal insertion. We propose that large structural variations involving non-coding DNA, similar to the CMTX3 mutation, may account for a proportion of the unsolved CMT cases.

## Materials and Methods

### Research participation

Participating family members gave informed consent according to the protocols approved by the Sydney Local Health District Human Ethics Review Committee, Concord Repatriation General Hospital, Sydney, Australia (reference number: HREC/11/CRGH/105).

### Genomic DNA extraction

Genomic DNA was extracted from peripheral blood using the PureGene Kit (Qiagen) following manufacturer’s instructions. Extractions were performed by Molecular Medicine Laboratory, Concord Repatriation General Hospital (Sydney, Australia).

### Whole genome sequencing

Genomic DNA samples (3 μg) were dispatched to NextCODE (Massachusetts, USA) who outsourced WGS of samples to Macrogen (South Korea). Paired-end (101 bp) sequencing was performed on a HiSeq 2000 sequencer (Illumina) following standard protocols.

### WGS bioinformatics analyses

Raw WGS data was returned to NextCODE who performed the following bioinformatics analyses. Access to all pipeline output files and visual representation of WGS data was made available through the Sequence Miner (NextCODE) application.

#### Sequence alignment

Sequence reads were aligned to the human reference sequence (hg19) using the Burrows-Wheeler Aligner (BWA) version 0.5.9 [54]. Alignments were merged into a single BAM file and marked for duplicates using Picard 1.55. Non-duplicate reads were selected for further downstream analyses.

#### Discordant paired end and split read detection

WGS data was assessed for discordant paired end reads and split reads using in house pipelines developed by NextCODE. For discordant paired end detection, scripts were developed to identify high quality read pairs mapping to different chromosomes or with inserts greater than 700 bp (more than twice the library mean insert size). Using a 200 bp window, the local maximum rearrangement position was identified and regions with generally poor read alignment were excluded. For split read detection, algorithms were used to extract reads whereby one half of the read mapped to the genome and the second half did not map locally.

### PCR amplification

Primers (X.F: 5’-CTCCAGCTTTGTTCTTTGGAC-3’; X.R: 5’-TCACCAACATTTCCAATCTCC-3’; 8.F: 5’-CAAACCCAATTCAGGTCCAG-3’; 8.R: 5’-GCCTAGGAGGTGTCCCTTTC-3’) were designed to amplify wild type chromosome X and the distal and proximal breakpoints of the 8q24.3 interchromosomal insertion. Multiplex PCR was performed in a 15 μl reaction containing 25 ng genomic DNA, 1X MyTaq Red Mix (Bioline), 8 pmol primer X.F, 8 pmol primer X.R, 2 pmol primer 8.F and 4 pmol primer 8.R. All PCR thermocycling was performed on an Eppendorf MasterCycler using a touchdown cycling protocol. Specific cycling temperatures are available on request. Amplicons were size fractionated on 1.5% (w/v) agarose gel at 40 V/cm. Amplified DNA was purified using the Isolate PCR and Gel Kit (Bioline) after gel electrophoresis following manufacturer’s instructions. Purified amplicons were submitted to Garvin Molecular Genetics (Sydney, Australia) for Sanger sequencing.

### Tissue culture of patient lymphoblasts

Patient EBV-transformed lymphoblast cell lines were prepared using standard procedures at Genetic Repositories Australia (Sydney, Australia). Sex and aged matched controls were obtained from the Genetic Repositories Australia. Lymphoblasts were maintained in RPMI 1640 (Invitrogen) supplemented with 10% fetal bovine serum (Scientifix) and 2 mM L-glutamine (Gibco).

### RNA isolation and cDNA synthesis

Total RNA was isolated from patient lymphoblast cells using Trizol (Life Technologies) according to the manufacturer’s instructions. RNA was eluted in 50 μl RNAse-free water, DNase-treated with Turbo DNase (Life Technologies) and stored at -80°C until required. RNA (1 μg) was converted to cDNA using iScript cDNA Synthesis Kit (Biorad) following manufacturer’s protocols.

### Gene expression analysis

Isolated cDNA (100 ng) was subjected to quantitative RT-PCR analysis using TaqMan Gene Expression Assays (Invitrogen) following manufacturer’s protocols. Quantitative RT-PCR was performed on a Step One Plus (Applied Biosystems) and relative fold difference was calculated using the comparative Ct method [55]. Target gene expression was determined relative to the housekeeping gene *18S*. For each RNA extraction (n = 3 per sample), quantitative RT-PCR reactions were performed in triplicate.

### Web resources

The 1000 Genomes Project, http://www.1000genomes.org

The 3D Genome Browser, http://promoter.bx.psu.edu/hi-c/index.html

dbSNP, http://www.ncbi.nlm.nih.gov/SNP/

NextCODE, https://www.nextcode.com

OMIM, http://www.omim.org

UCSC, https://genome.ucsc.edu

## Supporting Information

S1 Fig Sanger sequencing confirms the CMTX3 interchromosomal insertion breakpoint sequences as predicted by whole genome sequencing (WGS). Predicted sequence based on WGS data are shown on top and corresponding Sanger sequencing trace profile is displayed underneath for the proximal (A) and distal (B) breakpoints. WGS prediction data are color-coded blue for chromosome X sequence, orange for chromosome 8 sequence, and green for chromosome 12 sequence.
(TIF)

S2 Fig The CMTX3 interchromosomal insertion maps within a topological associated domain (TAD) on Xq27.1. Hi-C data from human embryonic stem cells [35] across the CMTX3 locus from chrX:13137,000,000–142,000,000 (top panel). Middle panel depicts the location of genes mapping within the locus, adapted from the UCSC Genome Browser. Dotted lines indicate the TAD boundaries based on the Hi-C data. Position of the CMTX3 insertion is indicated by the orange arrow. H3K27Ac marks, DNaseI hypersensitivity clusters and transcription factor ChIP-seq data from ENCODE are depicted (as visualized in UCSC Genome Browser) in the bottom panel.
(TIF)

S3 Fig *SOX3* mRNA expression is undetectable in patient and control lymphoblast cells. Real-time PCR amplification plot for *SOX3* (pink) and *18S* (green). The horizontal red line indicates the threshold value of fluorescence for calculating the Ct for *18S*.
(TIF)

## References

[1] Saporta ASD, Sottile SL, Miller LJ, Feely SME, Siskind CE, Shy ME. Charcot-Marie-Tooth disease subtypes and genetic testing strategies. Ann Neurol. 2011;69: 22–33. 10.1002/ana.22166 21280073 PMC3058597

[2] Drew AP, Zhu D, Kidambi A, Ly C, Tey S, Brewer MH, et al Improved inherited peripheral neuropathy genetic diagnosis by whole-exome sequencing. Mol Genet Genomic Med. 2015;3: 143–154. 10.1002/mgg3.126 25802885 PMC4367087

[3] Choi B-O, Koo SK, Park M-H, Rhee H, Yang S-J, Choi K-G, et al Exome sequencing is an efficient tool for genetic screening of Charcot-Marie-Tooth disease. Hum Mutat. 2012;33: 1610–1615. 10.1002/humu.22143 22730194

[4] Schabhüttl M, Wieland T, Senderek J, Baets J, Timmerman V, De Jonghe P, et al Whole-exome sequencing in patients with inherited neuropathies: outcome and challenges. J Neurol. 2014;261: 970–982. 10.1007/s00415-014-7289-8 24627108

[5] Ionasescu VV, Trofatter J, Haines JL, Summers AM, Ionasescu R, Searby C. Heterogeneity in X-linked recessive Charcot-Marie-Tooth neuropathy. Am J Hum Genet. 1991;48: 1075–1083. 1674639 PMC1683112

[6] Huttner IG, Kennerson ML, Reddel SW, Radovanovic D, Nicholson GA. Proof of genetic heterogeneity in X-linked Charcot-Marie-Tooth disease. Neurology. 2006;67: 2016–2021. 17159110 10.1212/01.wnl.0000247271.40782.b7

[7] Brewer MH, Changi F, Antonellis A, Fischbeck K, Polly P, Nicholson G, et al Evidence of a founder haplotype refines the X-linked Charcot-Marie-Tooth (CMTX3) locus to a 2.5 Mb region. Neurogenetics. 2008;9: 191–195. 10.1007/s10048-008-0126-4 18458969 PMC6852654

[8] Chaudhry R, Kidambi A, Brewer MH, Antonellis A, Mathews K, Nicholson G, et al Re-analysis of an original CMTX3 family using exome sequencing identifies a known BSCL2 mutation. Muscle Nerve. 2013;47: 922–924. 10.1002/mus.23743 23553728 PMC5175269

[9] Brewer MH. Molecular genetics of X-linked Charcot-Marie-Tooth neuropathy (CMTX3). Ph.D. Thesis, University of Sydney 2011 Available: http://opac.library.usyd.edu.au:80/record=b3855774~S4.

[10] Abecasis GR, Altshuler D, Auton A, Brooks LD, Durbin RM, Gibbs RA, et al A map of human genome variation from population-scale sequencing. Nature. 2010;467: 1061–1073. 10.1038/nature09534 20981092 PMC3042601

[11] Sherry ST, Ward MH, Kholodov M, Baker J, Phan L, Smigielski EM, et al dbSNP: the NCBI database of genetic variation. Nucleic Acids Res. 2001;29: 308–311. 11125122 10.1093/nar/29.1.308 PMC29783

[12] Raeymaekers P, Timmerman V, Nelis E, De Jonghe P, Hoogendijk JE, Baas F, et al Duplication in chromosome 17p11.2 in Charcot-Marie-Tooth neuropathy type 1a (CMT 1a). The HMSN Collaborative Research Group. Neuromuscul Disord. 1991;1: 93–97. 1822787 10.1016/0960-8966(91)90055-w

[13] Lupski JR, De Oca-Luna RM, Slaugenhaupt S, Pentao L, Guzzetta V, Trask BJ, et al DNA duplication associated with Charcot-Marie-Tooth disease type 1A. Cell. 1991;66: 219–232. 1677316 10.1016/0092-8674(91)90613-4

[14] Matsunami N, Smith B, Ballard L, Lensch MW, Robertson M, Albertsen H, et al Peripheral myelin protein-22 gene maps in the duplication in chromosome 17p11.2 associated with Charcot-Marie-Tooth 1A. Nat Genet. 1992;1: 176–179. 1303231 10.1038/ng0692-176

[15] Patel PI, Roa BB, Welcher AA, Schoener-Scott R, Trask BJ, Pentao L, et al The gene for the peripheral myelin protein PMP-22 is a candidate for Charcot-Marie-Tooth disease type 1A. Nat Genet. 1992;1: 159–165. 1303228 10.1038/ng0692-159

[16] Timmerman V, Nelis E, Van Hul W, Nieuwenhuijsen BW, Chen KL, Wang S, et al The peripheral myelin protein gene PMP-22 is contained within the Charcot-Marie-Tooth disease type 1A duplication. Nat Genet. 1992;1: 171–175. 1303230 10.1038/ng0692-171

[17] Valentijn LJ, Bolhuis PA, Zorn I, Hoogendijk JE, Van Den Bosch N, Hensels GW, et al The peripheral myelin gene PMP-22/GAS-3 is duplicated in Charcot-Marie-Tooth disease type 1A. Nat Genet. 1992;1: 166–170. 1303229 10.1038/ng0692-166

[18] Chance PF, Alderson MK, Leppig KA, Lensch MW, Matsunami N, Smith B, et al DNA deletion associated with hereditary neuropathy with liability to pressure palsies. Cell. 1993;72: 143–151. 8422677 10.1016/0092-8674(93)90058-x

[19] Huang J, Wu X, Montenegro G, Price J, Wang G, Vance JM, et al Copy number variations are a rare cause of non-CMT1A Charcot-Marie-Tooth disease. J Neurol. 2010;257: 735–741. 10.1007/s00415-009-5401-2 19949810 PMC2865568

[20] Høyer H, Braathen GJ, Eek AK, Nordang GBN, Skjelbred CF, Russell MB. Copy Number Variations in a Population-Based Study of Charcot-Marie-Tooth Disease. Biomed Res Int. 2015;2015: 1–7. 10.1155/2015/960404 PMC4306395 25648254

[21] Pehlivan D, Beck CR, Okamoto Y, Harel T, Akdemir ZHC, Jhangiani SN, et al The role of combined SNV and CNV burden in patients with distal symmetric polyneuropathy. Genet Med. 2015: 725–726. 10.1038/gim.2015.124 PMC5322766 26378787

[22] Høyer H, Braathen GJ, Eek AK, Skjelbred CF, Russell MB. Charcot-Marie-Tooth caused by a copy number variation in myelin protein zero. Eur J Med Genet. 2011;54: e580–e583. 10.1016/j.ejmg.2011.06.006 21787890

[23] Maeda MH, Mitsui J, Soong B-W, Takahashi Y, Ishiura H, Hayashi S, et al Increased gene dosage of myelin protein zero causes Charcot-Marie-Tooth disease. Ann Neurol. 2012;71: 84–92. 10.1002/ana.22658 22275255

[24] Ainsworth PJ, Bolton CF, Murphy BC, Stuart JA, Hahn AF. Genotype/phenotype correlation in affected individuals of a family with a deletion of the entire coding sequence of the connexin 32 gene. Hum Genet. 1998;103: 242–244. 9760211 10.1007/s004390050812

[25] Lin C, Numakura C, Ikegami T, Shizuka M, Shoji M, Nicholson G, et al Deletion and nonsense mutations of the connexin 32 gene associated with Charcot-Marie-Tooth disease. Tohoku J Exp Med. 1999;188: 239–244. 10587015 10.1620/tjem.188.239

[26] Nakagawa M, Takashima H, Umehara F, Arimura K, Miyashita F, Takenouchi N, et al Clinical phenotype in X-linked Charcot-Marie-Tooth disease with an entire deletion of the connexin 32 coding sequence. J Neurol Sci. 2001;185: 31–37. 11266688 10.1016/s0022-510x(01)00454-3

[27] Østern R, Fagerheim T, Hjellnes H, Nygård B, Mellgren SI, Nilssen Ø. Diagnostic laboratory testing for Charcot Marie Tooth disease (CMT): the spectrum of gene defects in Norwegian patients with CMT and its implications for future genetic test strategies. BMC Med Genet. 2013;14: 94 10.1186/1471-2350-14-94 24053775 PMC3849068

[28] Okamoto Y, Goksungur MT, Pehlivan D, Beck CR, Gonzaga-Jauregui C, Muzny DM, et al Exonic duplication CNV of NDRG1 associated with autosomal-recessive HMSN-Lom/CMT4D. Genet Med. 2013;16. 10.1038/gim.2013.155 PMC4224029 24136616

[29] Lupiáñez DG, Kraft K, Heinrich V, Krawitz P, Brancati F, Klopocki E, et al Disruptions of topological chromatin domains cause pathogenic rewiring of gene-enhancer interactions. Cell. 2015;161: 1012–1025. 10.1016/j.cell.2015.04.004 25959774 PMC4791538

[30] Weterman MaJ, Van Ruissen F, De Wissel M, Bordewijk L, Samijn JPA, Van Der Pol WL, et al Copy number variation upstream of PMP22 in Charcot-Marie-Tooth disease. Eur J Hum Genet. 2010;18: 421–428. 10.1038/ejhg.2009.186 19888301 PMC2987248

[31] Zhang F, Seeman P, Liu P, Weterman MaJ, Gonzaga-Jauregui C, Towne CF, et al Mechanisms for nonrecurrent genomic rearrangements associated with CMT1A or HNPP: rare CNVs as a cause for missing heritability. Am J Hum Genet. 2010;86: 892–903. 10.1016/j.ajhg.2010.05.001 20493460 PMC3032071

[32] Jones EA, Brewer MH, Srinivasan R, Krueger C, Sun G, Charney KN, et al Distal enhancers upstream of the Charcot-Marie-Tooth type 1A disease gene PMP22. Hum Mol Genet. 2012;21: 1581–1591. 10.1093/hmg/ddr595 22180461 PMC3298281

[33] Kleinjan DA, Van Heyningen V. Long-range control of gene expression: emerging mechanisms and disruption in disease. Am J Hum Genet. 2005;76: 8–32. 15549674 10.1086/426833 PMC1196435

[34] Spielmann M, Mundlos S. Structural variations, the regulatory landscape of the genome and their alteration in human disease. Bioessays. 2013;35: 533–543. 10.1002/bies.201200178 23625790

[35] Dixon JR, Selvaraj S, Yue F, Kim A, Li Y, Shen Y, et al Topological domains in mammalian genomes identified by analysis of chromatin interactions. Nature. 2012;485: 376–380. 10.1038/nature11082 22495300 PMC3356448

[36] Bowl MR, Nesbit MA, Harding B, Levy E, Jefferson A, Volpi E, et al An interstitial deletion-insertion involving chromosomes 2p25.3 and Xq27.1, near SOX3, causes X-linked recessive hypoparathyroidism. J Clin Invest. 2005;115: 2822–2831. 16167084 10.1172/JCI24156 PMC1201662

[37] Zhu H, Shang D, Sun M, Choi S, Liu Q, Hao J, et al X-linked congenital hypertrichosis syndrome is associated with interchromosomal insertions mediated by a human-specific palindrome near SOX3. Am J Hum Genet. 2011;88: 819–826. 10.1016/j.ajhg.2011.05.004 21636067 PMC3113246

[38] Destefano GM, Fantauzzo KA, Petukhova L, Kurban M, Tadin-Strapps M, Levy B, et al Position effect on FGF13 associated with X-linked congenital generalized hypertrichosis. Proc Natl Acad Sci U S A. 2013;110: 7790–7795. 10.1073/pnas.1216412110 23603273 PMC3651487

[39] Bunyan DJ, Robinson DO, Tyers AG, Huang S, Maloney VK, Grand FH, et al X-Linked Dominant Congenital Ptosis Cosegregating with an Interstitial Insertion of a Chromosome 1p21.3 Fragment into a Quasipalindromic Sequence in Xq27.1. Open Journal of Genetics. 2014;04: 415–425.

[40] Haines B, Hughes J, Corbett M, Shaw M, Innes J, Patel L, et al Interchromosomal insertional translocation at Xq26.3 alters SOX3 expression in an individual with XX male sex reversal. J Clin Endocrinol Metab. 2015;100: jc.2014-4383. 10.1210/jc.2014-4383 25781358

[41] Cotton AM, Avila L, Penaherrera MS, Affleck JG, Robinson WP, Brown CJ. Inactive X chromosome-specific reduction in placental DNA methylation. Hum Mol Genet. 2009;18: 3544–3552. 10.1093/hmg/ddp299 19586922 PMC2742397

[42] Smallwood PM, Munoz-Sanjuan I, Tong P, Macke JP, Hendry SH, Gilbert DJ, et al Fibroblast growth factor (FGF) homologous factors: new members of the FGF family implicated in nervous system development. Proc Natl Acad Sci U S A. 1996;93: 9850–9857. 8790420 10.1073/pnas.93.18.9850 PMC38518

[43] Weckselblatt B, Rudd MK. Human Structural Variation: Mechanisms of Chromosome Rearrangements. Trends Genet. 2015;31: 587–599. 10.1016/j.tig.2015.05.010 26209074 PMC4600437

[44] Carvalho CMB, Lupski JR. Mechanisms underlying structural variant formation in genomic disorders. Nature reviews Genetics. 2016;17: 224–238. 10.1038/nrg.2015.25 26924765 PMC4827625

[45] Hastings PJ, Ira G, Lupski JR. A microhomology-mediated break-induced replication model for the origin of human copy number variation. PLoS Genet. 2009;5: e1000327 10.1371/journal.pgen.1000327 19180184 PMC2621351

[46] Onozawa M, Zhang Z, Kim YJ, Goldberg L, Varga T, Bergsagel PL, et al Repair of DNA double-strand breaks by templated nucleotide sequence insertions derived from distant regions of the genome. Proc Natl Acad Sci U S A. 2014;111: 7729–7734. 10.1073/pnas.1321889111 24821809 PMC4040595

[47] Kurahashi H, Inagaki H, Ohye T, Kogo H, Kato T, Emanuel BS. Palindrome-mediated chromosomal translocations in humans. DNA repair. 2006;5: 1136–1145. 16829213 10.1016/j.dnarep.2006.05.035 PMC2824556

[48] Zhang F, Khajavi M, Connolly AM, Towne CF, Batish SD, Lupski JR. The DNA replication FoSTeS/MMBIR mechanism can generate genomic, genic and exonic complex rearrangements in humans. Nat Genet. 2009;41: 849–853. 10.1038/ng.399 19543269 PMC4461229

[49] Sakofsky CJ, Ayyar S, Deem AK, Chung WH, Ira G, Malkova A. Translesion Polymerases Drive Microhomology-Mediated Break-Induced Replication Leading to Complex Chromosomal Rearrangements. Mol Cell. 2015;60: 860–872. 10.1016/j.molcel.2015.10.041 26669261 PMC4688117

[50] Belkadi A, Bolze A, Itan Y, Cobat A, Vincent QB, Antipenko A, et al Whole-genome sequencing is more powerful than whole-exome sequencing for detecting exome variants. Proc Natl Acad Sci U S A. 2015: 1418631112-. 10.1073/pnas.1418631112 PMC4418901 25827230

[51] Zhang Y, Haraksingh R, Grubert F, Abyzov A, Gerstein M, Weissman S, et al Child Development and Structural Variation in the Human Genome. Child Dev. 2013;84: 34–48. 10.1111/cdev.12051 23311762

[52] Redon R, Ishikawa S, Fitch KR, Feuk L, Perry GH, Andrews TD, et al Global variation in copy number in the human genome. Nature. 2006;444: 444–454. 17122850 10.1038/nature05329 PMC2669898

[53] Feuk L, Carson AR, Scherer SW. Structural variation in the human genome. Nat Rev Genet. 2006;7: 85–97. 16418744 10.1038/nrg1767

[54] Li H, Durbin R. Fast and accurate short read alignment with Burrows-Wheeler transform. Bioinformatics (Oxford, England). 2009;25: 1754–1760. 10.1093/bioinformatics/btp324 PMC2705234 19451168

[55] Livak KJ, Schmittgen TD. Analysis of relative gene expression data using real-time quantitative PCR and the 2(-Delta Delta C(T)) Method. Methods. 2001;25: 402–408. 11846609 10.1006/meth.2001.1262

