# Supplementary figures and images for: Whole Genome Sequencing Identifies a 78 kb Insertion from Chromosome 8 as the Cause of Charcot-Marie-Tooth Neuropathy CMTX3
Source: PLoS Genet. 2016 Jul 20;12(7):e1006177. doi: 10.1371/journal.pgen.1006177 (PMC4954712; doi:10.1371/journal.pgen.1006177)

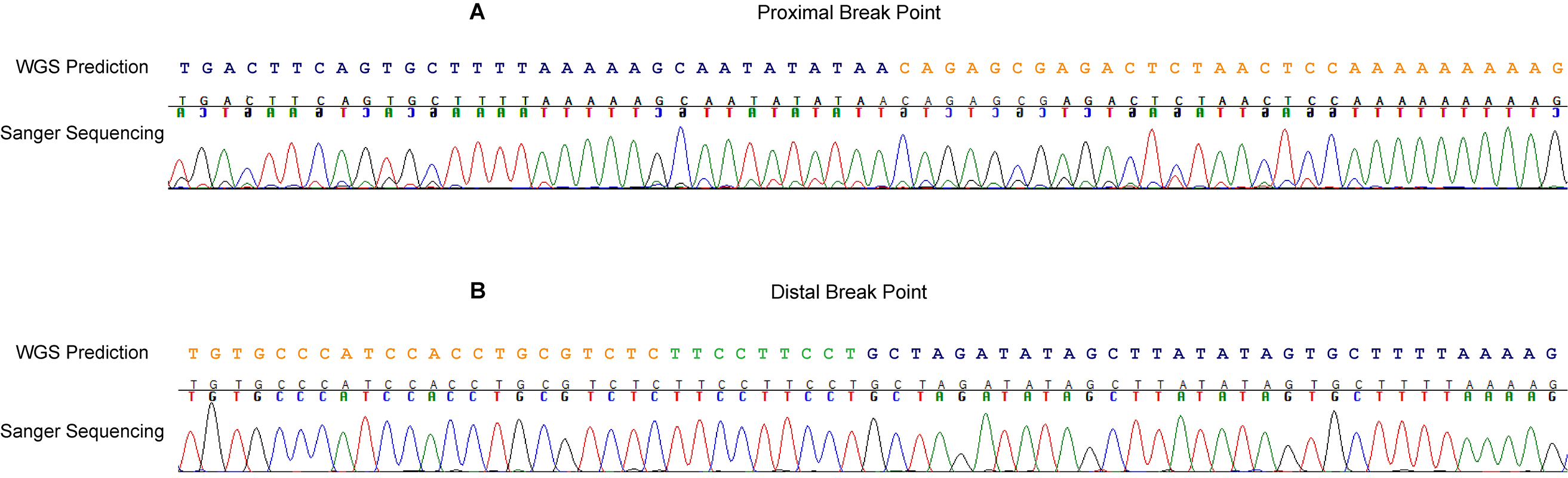

Supplement: S1 Fig — Predicted sequence based on WGS data are shown on top and corresponding Sanger sequencing trace profile is displayed underneath for the proximal (A) and distal (B) breakpoints. WGS prediction data are color-coded blue for chromosome X sequence, orange for chromosome 8 sequence, and green for chromosome 12 sequence. (TIF) [file pgen.1006177.s001.tif]

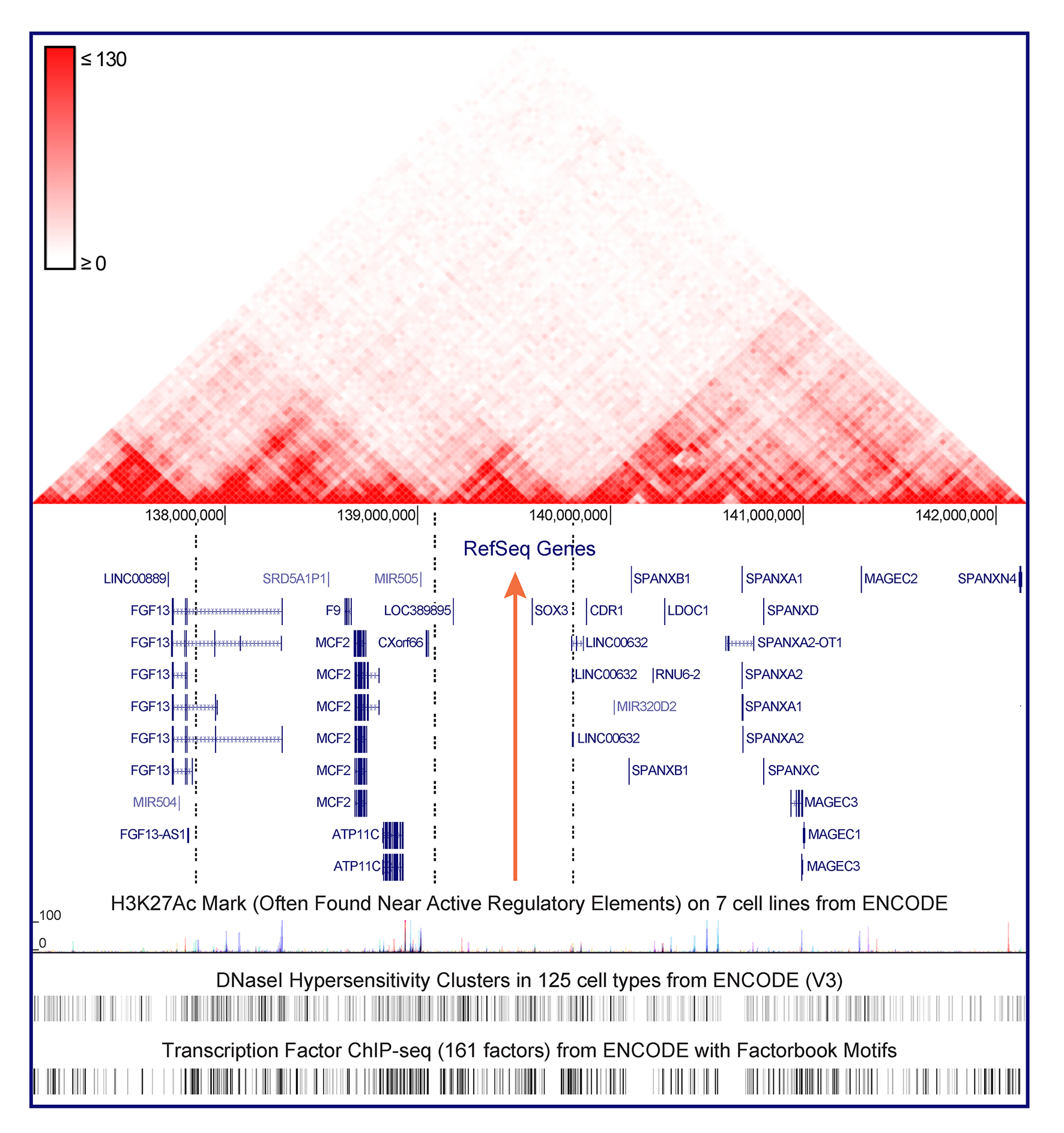

Supplement: S2 Fig — Hi-C data from human embryonic stem cells [35] across the CMTX3 locus from chrX:13137,000,000–142,000,000 (top panel). Middle panel depicts the location of genes mapping within the locus, adapted from the UCSC Genome Browser. Dotted lines indicate the TAD boundaries based on the Hi-C data. Position of the CMTX3 insertion is indicated by the orange arrow. H3K27Ac marks, DNaseI hypersensitivity clusters and transcription factor ChIP-seq data from ENCODE are depicted (as visualized in UCSC Genome Browser) in the bottom panel. (TIF) [file pgen.1006177.s002.tif]

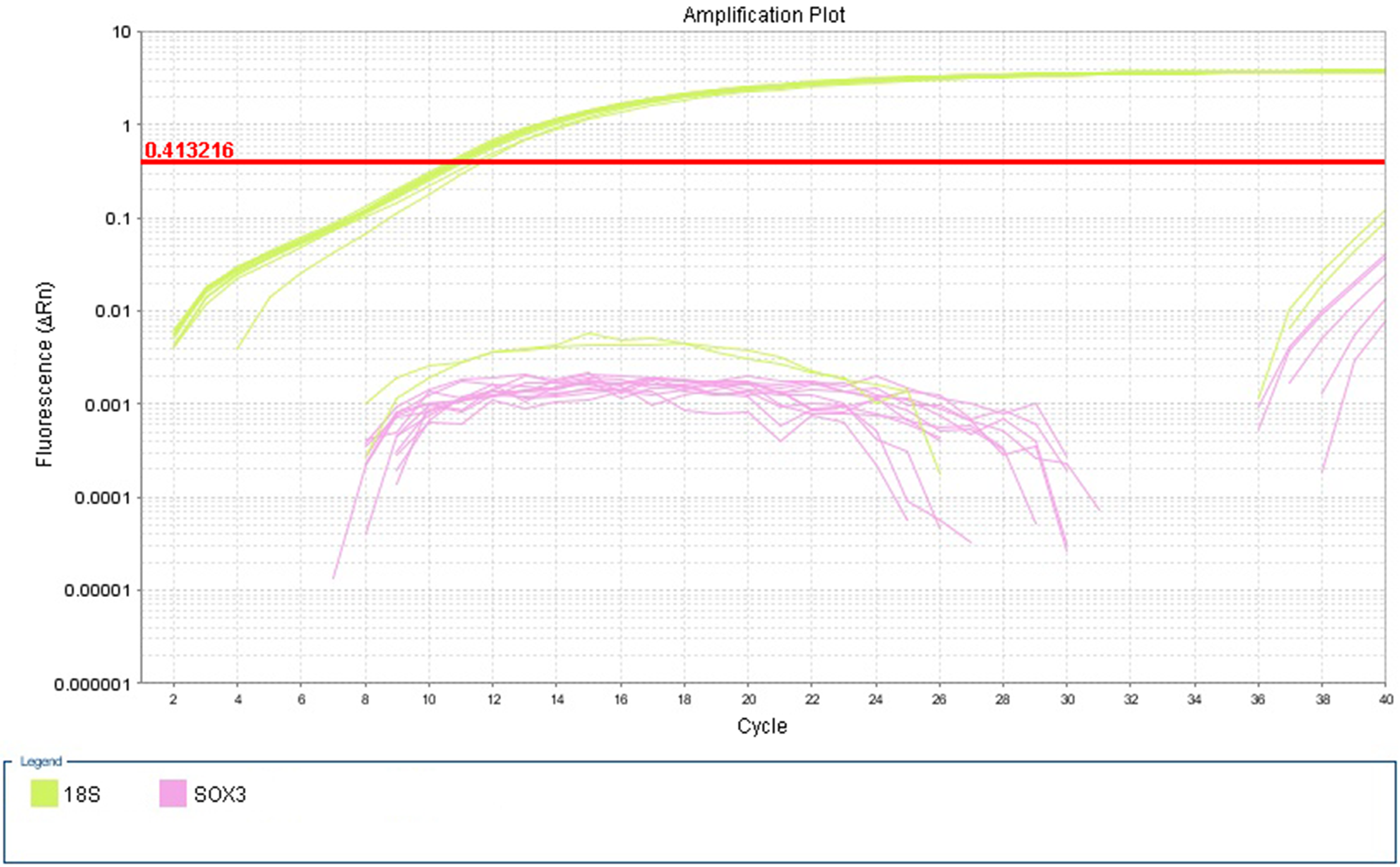

Supplement: S3 Fig — Real-time PCR amplification plot for SOX3 (pink) and 18S (green). The horizontal red line indicates the threshold value of fluorescence for calculating the Ct for 18S. (TIF) [file pgen.1006177.s003.tif]
